# Reframing patient complaints as a quality and governance function in healthcare

**DOI:** 10.1093/intqhc/mzag073

**Published:** 2026-05-22

**Authors:** Judit Pripkó, Henriette Pusztafalvi

**Affiliations:** Faculty of Health Sciences, Doctoral School of Health Sciences, University of Pécs, Pécs, 7621, Hungary; Faculty of Health Sciences, Institute of Health Insurance, Department of Health Promotion and Public Health, University of Pécs, Pécs, 7621, Hungary

## Perspective

Patient complaints are commonly treated as administrative tasks within healthcare organisations, focused on documentation, investigation, and procedural response. Evidence shows that complaints frequently arise from failures in communication, coordination, and responsiveness rather than purely technical deficiencies in care delivery [1–4].

Complaints should therefore not be understood solely as expressions of dissatisfaction, but as structured signals reflecting how healthcare processes are experienced and interpreted by patients. Unlike conventional performance indicators, which capture measurable clinical outcomes, complaints provide insight into dimensions such as information flow, relational trust, and perceived responsiveness. These dimensions are central to patient-centred care but remain insufficiently integrated into formal quality measurement systems [5, 6].

Despite this potential, complaint handling is typically organised around administrative processes that prioritize case resolution over system-level learning. Complaints are recorded, investigated, and closed, but the information they contain is rarely aggregated or systematically integrated into quality governance structures. As a result, their contribution to organizational decision-making and improvement remains limited [1, 7].

This gap points to the need for a conceptual shift: complaints can be understood as system-level signals revealing misalignment between intended, delivered, and experienced care. On this basis, complaints should be repositioned within healthcare quality governance as sources of actionable intelligence.

In most healthcare systems, complaint handling follows an administrative logic centred on individual case resolution. Infor­mation remains case-bound, categorization is inconsistent, and aggregation is not systematically embedded in quality management processes. Consequently, recurrent issues—particularly those related to communication and coordination—may remain unrecognised or only identified retrospectively [2–4, 8].

A governance-oriented approach rests on a different organisational logic. Complaints need to be handled as structured inputs into system-level processes. This requires more than documenting individual cases: complaints must be standardized, classified, and aggregated across units and over time, so that patterns can be reviewed at governance level. In this model, complaints become part of a broader information architecture, enabling organisations to identify vulnerabilities not captured by traditional metrics [5, 9].

The distinction between these approaches is functional rather than procedural. In administrative handling, the endpoint is case closure; in governance-integrated handling, the endpoint is organisational learning and system adjustment. This shift requires explicit mechanisms linking complaint data to decision-making processes, including criteria for escalation, regular governance review, and translation of identified patterns into improvement actions. Without such mechanisms, calls to ‘use complaints more effectively’ remain normative rather than operational.

**Figure 1:**
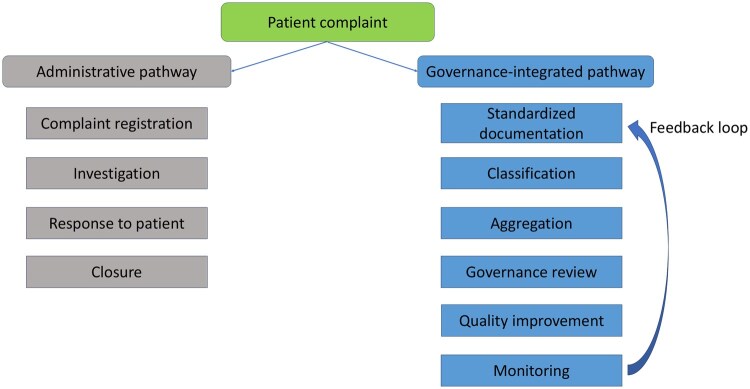
Conceptual framework of administrative and governance-integrated complaint pathways, illustrating inputs, processes, outputs, and feedback loops.

This difference is conceptualised in Fig. 1, which illustrates administrative and governance-integrated pathways of patient complaint handling. The administrative pathway represents a linear process from registration to closure, whereas the governance-integrated pathway embeds complaints in a cyclical process ­supported by feedback loops. An operational comparison of the two approaches is provided in Supplementary Table S1.

Within this framework, standardization is a critical enabling condition. Without shared documentation and classification, complaints remain narrative accounts that are difficult to compare or aggregate. With standardization, recurring patterns—particularly in communication, access, and coordination—become visible and analysable at the system level [2–4, 8].

Aggregation marks the transition from case management to system analysis. Routine aggregation enables organisations to move beyond isolated incidents and recognise recurring patterns and underlying vulnerabilities. This is particularly relevant for communication-related problems, which often appear as repeated low-level concerns rather than single critical events.

For these signals to influence organisational behaviour, they must be embedded in governance structures. Regular review of aggregated complaint data within quality and safety forums enables alignment with other organizational data sources, supporting prioritization and targeted improvement actions [1, 9]. Escalation should depend not only on severity but also on recurrence and system-level relevance.

Linking analysis to action requires explicit integration with quality improvement processes. Complaint-derived insights should trigger defined responsibilities, action plans, and timelines. Monitoring is then needed to assess whether interventions reduce recurrence and improve patient experience, thereby closing the loop between detection and response.

A defining feature of the governance-integrated model is the presence of feedback loops. Beyond responding to individual complainants, feedback also operates at the organizational level by informing practice and supporting system-level learning. Complaint management thus becomes an adaptive component of healthcare governance, continuously shaped by the interaction between patient input and organisational response. Importantly, this does not require new systems, but the reconfiguration of existing processes to generate actionable information for quality improvement.

Taken together, this perspective advances the field by moving beyond the recognition that complaints are valuable, towards specifying how they can be operationalized within governance systems. This article outlines a structured conceptual approach for translating that value into system-level learning [5, 6, 10].

It is important to note that the framework presented is conceptual in nature. While it outlines how complaint data can be structured and integrated into governance processes, its implementation in real-world healthcare organizations is likely to be challenging. This is due to variability in organizational capacity, data infrastructure, and the availability of transparent and valid data to support reliable classification, aggregation, and interpretation.

Moreover, there are currently limited empirical examples of healthcare organizations applying complaint data in a systematic, governance-integrated manner. As such, the model should be understood as a structured approach that requires further validation.

Future research should therefore focus on empirical studies and case descriptions that examine how organizations operationalize complaint-based learning, including barriers, enabling factors, data quality considerations, and measurable impacts on quality improvement.

Integrating complaint data into governance strengthens the capacity to detect recurrent problems in communication, coordi­nation, and responsiveness—domains central to patient safety yet often underrepresented in traditional metrics [2–4, 8]. At the same time, it reinforces accountability by linking patient-reported concerns to formal decision-making processes [1, 9].

In conclusion, patient complaints should be recognized as strategic resources for healthcare quality improvement rather than administrative burdens. Embedding complaint management within governance structures enables organizations to translate patient experiences into system-level insights, supporting earlier risk identification, more targeted interventions, and stronger alignment between delivered and experienced care.

The administrative pathway represents a linear process focused on case resolution, whereas the governance-integrated pathway embeds complaints in a cyclical process of documentation, classification, aggregation, review, improvement, and monitoring, supported by feedback loops.

## Supplementary Material

mzag073_Supplementary_Data

## References

[1] van Dael J , Reader TW, Gillespie A et al Learning from complaints in healthcare: a realist review of academic literature, policy evidence and front-line insights. BMJ Qual Saf 2020; 29:684–95. 10.1136/bmjqs-2019-009704 PMC7398301 32019824

[2] Hult A , Lundgren E, Fröjd C et al Patient complaints about communication in cancer care settings: hidden between the lines. Patient Educ Couns 2023;114:107838. 10.1016/j.pec.2023.107838 37295042

[3] Skär L , Söderberg S. Patients’ complaints regarding health­care encounters and communication. Nurs Open 2018;5:224–32. 10.1002/nop2.132 29599998 PMC5867282

[4] Murphy F , O’Donnell H, O’Doherty J. A comparative analysis of patient complaints in Ireland and the UK. Int J Qual Health Care 2023;34:mzac037. 10.1093/intqhc/mzac037

[5] Doyle C , Lennox L, Bell D. A systematic review of evidence on the links between patient experience and clinical safety and effectiveness. BMJ Open. 2013;3:e001570. 10.1136/bmjopen-2012-001570 PMC3549241 23293244

[6] Jangland E , Gunningberg L, Carlsson M. Patients’ and relatives’ complaints about encounters and communication in health care: evidence for quality improvement. Patient Educ Couns 2009;75:199–204. 10.1016/j.pec.2008.10.007 19038522

[7] Beaupert F , Carney T, Chiarella M et al Regulating healthcare complaints: a literature review. Int J Health Care Qual Assur 2014;27:505–18. 10.1108/IJHCQA-05-2013-0053 25115053

[8] Pripkó J. Az egészségügyben megjelenő betegpanaszok vizsgálata a bioetikai alapelvek mentén. Tanulmányok 2025;1:115–28.

[9] Reader TW , Gillespie A. Patient neglect in healthcare institutions: a systematic review and conceptual model. Soc Sci Med 2013;95:70–8. 10.1016/j.socscimed.2013.07.010 PMC3660245 23631468

[10] Bleich SN , Özaltin E, Murray CJL. How does satisfaction with the healthcare system relate to patient experience? Bull World Health Organ 2009;87:271–8. 10.2471/BLT.07.050401 19551235 PMC2672587

