# Supplementary material for: Reframing patient complaints as a quality and governance function in healthcare
Source: Int J Qual Health Care. 2026 May 22;38(2):mzag073. doi: 10.1093/intqhc/mzag073 (PMC13235711; doi:10.1093/intqhc/mzag073)
Supplement: mzag073_Supplementary_Data [file mzag073_supplementary_data.docx]

Table 1. Operational comparison of administrative and governance-integrated complaint handling

| **Component** | **Administrative approach** | **Governance-integrated approach** | **Operational implication** |
| --- | --- | --- | --- |
| Complaint registration | Recorded for documentation and response. | Recorded as structured data for analysis and learning. | Include fields for care setting, complaint type, severity, recurrence, and responsible unit. |
| Standardized documentation | Narrative, variable across units. | Standardized, comparable across cases and time. | Apply a shared taxonomy (e.g. communication, access, coordination, safety). |
| Classification | Used for routing and case handling. | Used to identify system functions and underlying issues. | Distinguish between individual error and system-level vulnerability. |
| Investigation | Focus on the individual case and its resolution. | Links individual cases to patterns and recurrent issues. | Enable flagging of cases with system relevance. |
| Aggregation and analysis | Limited aggregation; mainly case-level data. | Routine aggregation to detect trends and weak signals. | Review complaint data periodically within quality systems. |
| Escalation | Triggered by severity or legal risk. | Triggered by recurrence, system risk, or cross-unit relevance. | Define criteria for escalation to management or governance level. |
| Governance review | Managed within administrative or legal functions. | Integrated into quality and governance structures. | Include complaint data in governance meetings alongside clinical indicators. |
| Quality improvement | Actions limited to individual cases. | Patterns trigger system-level improvement actions. | Assign responsibility, action plans, and timelines for each issue. |
| Monitoring | Focus on response time and closure. | Evaluates impact of improvement and recurrence. | Track trends, repeated complaints, and outcomes of actions. |
| Feedback | Response provided to the complainant. | Feedback at individual and organisational level. | Ensure communication to patients and internal learning loops. |
